# White matter hyperintensities and neurodegenerative dementias

**DOI:** 10.18632/aging.101967

**Published:** 2019-05-18

**Authors:** Celeste Sassi

**Affiliations:** 1 Department of Neurology, Experimental Neurology, Charité Universitätsmedizin Berlin, Berlin, Germany

**Keywords:** white matter hyperintensities (WMHs), leukodystrophies, dementia, brain hypoperfusion, microglia

White matter hyperintensities (WMHs), to different extents and diagnostic importance, are a common feature of different neurodegenerative dementias. In very rare Mendelian leukodystrophies, such as polycystic lipomembranous osteodysplasia with sclerosing leukoencephalopathy (PLOSL) and hereditary diffuse leukoencephalopathy with spheroids (HDLS), WMHs represent essential diagnostic features. Whereas WMHs are emerging now as potential biomarker for Alzheimer’s disease (AD), from the common late-onset sporadic cases to the autosomal dominant ones, where white matter microstructural abnormalities are identifiable between 5 and 10 years before the onset of symptoms [1]. However, whether WMHs play a critical role in the dementia pathogenic cascade or are simply incidental and whether there is a convergent mechanism linking WMHs and diverse neurodegenerative dementias remains controversial.

PLOSL and HDLS are caused by autosomal recessive mutations in triggering receptor expressed on myeloid cells 2 (*TREM2*) and TYRO protein tyrosine kinase-binding protein (*TYROBP*) and autosomal dominant mutations in the tyrosine kinase domain of colony stimulating factor 1 receptor (*CSF1R*), respectively. These represent critical microglial cellular receptors (TREM2 and CSF1R) or transmembrane protein (TYROBP). Very rare heterozygous mutations in these same genes have been associated to relatively more common dementias such as familial frontotemporal dementia (FTD)-like and sporadic AD [2–4], sharing with PLOSL and HDLS, to a variable degree, clinical presentation, characterized by progressive cognitive impairment, stroke-like episodes, epilepsy, migrane and pyramidal and extrapyramidal signs. Moreover, although granulin (*GRN)* has not been identified as Mendelian leukodystrophy causative gene, the clinical features of FTD caused by *GRN* mutations significantly overlap with HDLS, although with a milder progression, generally later onset and less frequent involvement of deep white matter. This may be likely due to the different cellular function and subcellular localization within microglial cells of progranulin (PGRN), encoded by *GRN*. Unlike TREM2 and CSF1R, critical microglial cellular receptors, PGRN is a secretory lysosomal protein playing a pivotal role in regulating lysosomal function. Although WMHs are not a common finding in FTD caused by TAR DNA-binding protein 43, tau and fused in sarcoma pathology, these have been reported significantly associated to FTD patients with *GRN* mutations and may represent a biomarker to distinguish different FTD subtypes [5]. Analogously to the shared clinical features, the location, progression and extension of the WMHs in patients carrying *TREM2*, *TYROBP*, *CSF1R* and *GRN* pathogenic mutations display a significant overlap and mainly affect deep periventricular white matter, centrum semiovale, whereas WMHs or white matter microstructural changes in patients with autosomal dominant AD have been detected in medial frontal and posterior parietal-orbital white matter. Interestingly, these areas are located at the border zones between 2 different arterial supplies: middle prefrontal areas are supplied by terminal branches of anterior cerebral artery (ACA) and middle cerebral artery (MCA); posterior parietal areas are supplied by terminal branches of MCA and posterior cerebral artery (PCA). In addition, deep white matter areas such as periventricular areas and centrum semiovale are irrorated by MCA deep penetrating arterioles that already at 50 years of age display tortuosity and increased vascular resistance. Thus, these white matter regions are particularly vulnerable to watershed ischemic lesions during hypoperfusion [6]. Importantly, although severe vascular risk factors have been ruled out in the study of patients with neurodegenerative dementias displaying WMHs, hypoperfusion has been reported in FTD patients with *GRN* mutations and PLOSL patients and stroke-like episodes and migrane may represent a consequence of a reduced cerebral perfusion. Finally, different degrees of brain hypoperfusion-ischemia in stroke experimental models are responsible for the rapid recruitment of microglia that exert a protective function, contributing to the ischemic lesion resolution and may influence angiogenesis-vasculogenesis during embryogenesis and diseases [7]. Possibly, hypoperfusion may be a direct consequence of germline mutations in critical microglia genes or a preexistent hypoperfusion may unmask a microglia impairment due to *de novo* mutations in *TREM2*, *TYROBP*, *CSF1R*, *GRN* or simply aging. Therefore, before completely excluding vascular factors in the pathogenesis of early-onset WMHs in neurodegenerative dementias, it may be worth investigating the link between microglia and brain perfusion.
